# Serum NSE and S100B protein levels for evaluating the impaired consciousness in patients with acute carbon monoxide poisoning

**DOI:** 10.1097/MD.0000000000026458

**Published:** 2021-06-25

**Authors:** Litao Zhang, Jing Zhao, Qingqing Hao, Xin Xu, Hu Han, Jianguo Li

**Affiliations:** a Department of Emergency; b Department of Oncology; c Department of Cardiology, Hebei General Hospital, Shijiazhuang, Hebei, China.

**Keywords:** acute carbon monoxide poisoning, consciousness impairment, neuron-specific enolase, S100B

## Abstract

The aim of this study was to investigate the associations between the levels of neuron-specific enolase (NSE) and S100B protein and coma duration, and evaluate the optimal cut-off values for prediction coma duration ≥ 72 hours in patients with acute carbon monoxide poisoning (ACOP).

A total of 60 patients with ACOP were divided into 3 following groups according to their status of consciousness and coma duration at admission: Awake group [Glasgow Coma Scale score (GCS score) ≥ 13 points], Coma < 72 hours group (GCS score < 13 points and coma duration < 72 h), and Coma ≥ 72 hours group (GCS score < 13 points and coma duration ≥ 72 h). The levels of serum NSE and S100B protein were measured after admission.

There were significant differences in GCS score, carbon monoxide (CO) exposure time, NSE, and S100B levels between the Coma ≥ 72 h group and the Awake group, and between the Coma < 72 h group and the Awake group. Significant differences in GCS score, NSE, and S100B levels were also found between Coma ≥ 72 h group and Coma < 72 h group. Correlation analysis showed that NSE and S100B were positively correlated (*r*_s_ = 0.590, *P* < .01); NSE and S100B were negatively correlated with GCS score (*r*_s_ = -0.583, *r*_s_ = -0.590, respectively, both *P* < .01). The areas under the curve (AUCs) of NSE, S100B, and GCS score to predict the coma duration ≥ 72 hours were 0.754, 0.791, and 0.785, respectively. Pairwise comparisons did not show differences among the 3 groups (all *P* > .05). The sensitivity and specificity of NSE prediction with a cut-off value of 13 μg/L were 80% and 64%, respectively, and those of S100B prediction with a cut-off value of 0.43 μg/L were 70% and 88%, respectively.

The NSE and S100B protein levels were significantly correlated with the degree of impaired consciousness and had the same clinical value in predicting coma duration of ≥ 72 hours in patients with ACOP.

## 1 Introduction

Acute carbon monoxide poisoning (ACOP) is a common acute disease in China. It is one of the most common toxic diseases in emergency medicine, with high mortality and disability rate. Its main clinical manifestations include headaches, fatigue, cherry red lips, confusion, collapse, and coma. It can also lead to multiple organ diseases, while neurologic sequelae are the most frequent form of morbidity. The cerebral cortex, globus pallidus, and internal capsule are the commonly affected tissues. The formation of carboxyhemoglobin reduces the blood's oxygen-carrying capacity, causing tissue hypoxia and leading to ischemic stroke.^[1]^ ACOP can also lead to delayed encephalopathy after ACOP (DEACMP).^[2,3]^ Coma duration is one of the objective predictors of poor prognosis in emergency patients with ACOP. Therefore, evaluating the degree of consciousness impairment in patients with ACOP, especially the duration, is crucial to understand the patient's condition, prevent related complications, and evaluate patients’ prognosis.

Serum neuron-specific enolase (NSE) and S100B protein are commonly used biomarkers to evaluate central nervous system (CNS) injury. However, so far, only a few studies evaluated the correlation between serum NSE levels and consciousness impairment after carbon monoxide (CO) exposure, reporting inconsistent results.^[4]^ S100B protein is associated with glial cell activation and DEACMP occurrence in patients with ACOP.^[5]^Also, some studies have evaluated the S100B level as an indicator of the severity of CNS affection in patients with ACOP.^[6,7]^ However, early prediction of coma duration in patients with ACOP remains unclear.

In this study, we investigated the associations between the levels of NSE and S100B protein and coma duration, and evaluated the optimal cut-off values for prediction coma duration ≥ 72 hours in patients with ACOP.

## 2 Materials and methods

### 2.1 Study subjects

A total of 60 ACOP patients (including 30 males and 30 females, aged 50.63 ± 16.00 years) from the Emergency Department of Hebei General Hospital were enrolled from January 2019 to December 2019. ACOP was diagnosed by the emergency physician based on clinical history, CO poisoning associated clinical symptoms and signs, and carboxyhemoglobin (COHb) levels.^[8]^ Inclusion criteria were age ≥ 18 years, patients admitted within 24 hours after onset; and with complete medical data. Exclusion criteria were patients with malignant tumor, primary immunodeficiency, or immunosuppressant therapy; patients with previous cerebrovascular sequelae with impaired consciousness, decompensated cirrhosis, hereditary diseases, congenital metabolic diseases, or the end stage of other chronic diseases with organ dysfunction; patients with brain injury (head trauma, cerebral stroke, intracranial infection, epilepsy, and so on); patients with cardiac arrest and return of spontaneous circulation; and patients who died within 72 hours of treatment after admission or discharged.

### 2.2 Methods

The COHb levels were measured immediately after the patients’ consciousness status was evaluated using a Glasgow Coma Scale score (GCS score). Coma was defined as a GCS score < 13 points; being awake was defined as GCS score ≥ 13 points. Patients with artificial airways should be scored with 5–3–1, where 5 points indicated good orientation, 1 point indicated nonresponse, and 3 points indicated the middle status.

Within 24 hours after admission, 5 mL of venous blood was collected and centrifuged for 20 minutes at 1000 r/min. The serum was separated, and the protein level was determined by NSE and S100B protein enzyme-linked immunosorbent assay kit (provided by Wuhan Elabscience Biotechnology Co., Ltd). Coma duration was defined as the length of time (hours) from establishing the patient was in a comatose state to waking up. The duration of CO exposure was defined as the time (hours) of exposure to CO estimated by the patient's medical history.

After entering the emergency department, all patients were given routine treatment, including inhaling oxygen, drugs for preventing and treating brain edema, improving brain metabolism, anti-oxygen free radicals, and promoting awakening. Nutritional and organ function support, including tracheal intubation, ventilator-assisted breathing, circulatory support, were given when necessary. Hyperbaric oxygen therapy should be performed as soon as possible, once or twice a day. According to the status of consciousness and coma duration, the patients were divided into 3 groups: Awake group (GCS score ≥ 13 points), Coma < 72 h group (GCS score < 13 points and coma duration < 72 h), Coma ≥ 72 h group (GCS score < 13 points and coma duration ≥ 72 h).

### 2.3 Statistical analysis

The data were processed and analyzed by SPSS 26.0 software. The measurement data with normal distribution were expressed by mean ± standard deviation ($\bar{X}\pm S$). One-way analysis of variance (One-way ANOVA) or Welch test was used to compare the measurement data among groups, and Tamhane T2 test was used for pairwise comparison. The continuous variables with non-normal distribution were represented by median (quartile) [M (Q_L_, Q_U_)], and the nonparametric Kruskal--Wallis rank-sum test was used for comparisons among multiple groups. The correlation between the 2 groups of measurement data was analyzed by Spearman correlation analysis. The counting data were expressed by frequency and rate, and the comparison between groups was performed by χ^2^ test.

MedCalc 12.7.0 software was used to calculate the receiver operating characteristic (ROC) curve and the area under the curve (AUC). AUC of 0.8 to 0.9 represented the optimal prediction accuracy, more than 0.7 had clinical value, and less than 0.7 had the poor predictive ability. Z test was used to compare the 2 AUCs. The Youden index was calculated, and the score at the maximum Youden index was taken as the cut-off value. The sensitivity, specificity, positive likelihood ratio, and negative likelihood ratio were determined according to the cut-off value. *P* < .05 was considered to be statistically significant.

## 3 Results

### 3.1 General data of study subjects

Patients were divided into 3 groups: Awake group (15 cases), Coma <72 h group (25 cases), and Coma ≥ 72 h group (20 cases). There was no significant difference in age and gender among the 3 groups (all *P* > .05) **(**Table 1).

**Table 1:** General data of patients [$\bar{X}\pm S$, n (%)].

	Awake group (n = 15)	Coma < 72h group (n = 25)	Coma ≥ 72h group (n = 20)	χ2/F	*P*
Age	56.07 ± 19.93	50.84 ± 18.65	49.15 ± 21.02	0.554	.578
Males	9 (60)	11 (44)	10 (50)	0.960	.619

### 3.2 Comparison of data among patients in the three groups

There was no significant difference in COHb among the 3 groups (*P *> .05). There were significant differences in GCS score, CO exposure time, NSE, and S100B levels between the Coma ≥ 72 h group and the Awake group (all *P* < .01). There were also significant differences between the Coma < 72 h group and the Awake group (all *P* < .01). There were significant differences in GCS score, NSE, and S100B levels between Coma ≥ 72 h group and Coma < 72 h group (all *P* < .05) (Table 2).

**Table 2:** Comparison of related data among 3 groups of patients.

Groups	Number of cases	COHb	GCS score	CO exposure duration, h	NSE, μg/L	S100B, μg/L
Awake group	15	0.12 ± 0.44	14 (13,15)	8.27 ± 1.67	6.74 ± 1.38	0.17 ± 0.05
Coma < 72 h group	25	0.15 ± 0.04	7 (6,10)^∗^	10.80 ± 2.42^∗^	12.24 ± 3.58^∗^	0.32 ± 0.14^∗^
Coma ≥ 72 h group	20	0.15 ± 0.05	5 (4,6)^∗^^,^^†^	11.80 ± 2.12^∗^	16.65 ± 5.69^∗^^,^^†^	0.53 ± 0.24^∗^^,^^†^
F/H		3.01	39.73	11.96	45.35	28.10
*P*		.057	.000	.000	.000	.000

Compared with awake group.

∗ *P *< .01; compared with coma <72 h group.

† *P *< .05.

### 3.3 Correlation analysis of NSE, S100B, and GCS scores

**Figure 1:**
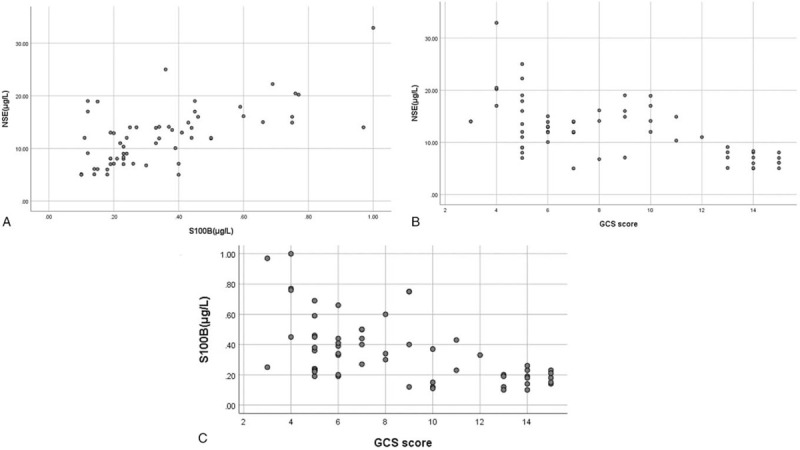
Correlation between S100B (A), NSE (B), and GCS (C) score in acute carbon monoxide poisoning.

NSE and S100B was positively correlated (*r*_s_ = 0.590, *P* < .01), GCS score was negatively correlated with NSE and S100B, respectively (*r*_s_ = −0.583 and *r*_s_ = −0.590, respectively, both *P* < .01) (Fig. 1).

### 3.4 ROC curve analysis

**Figure 2:**
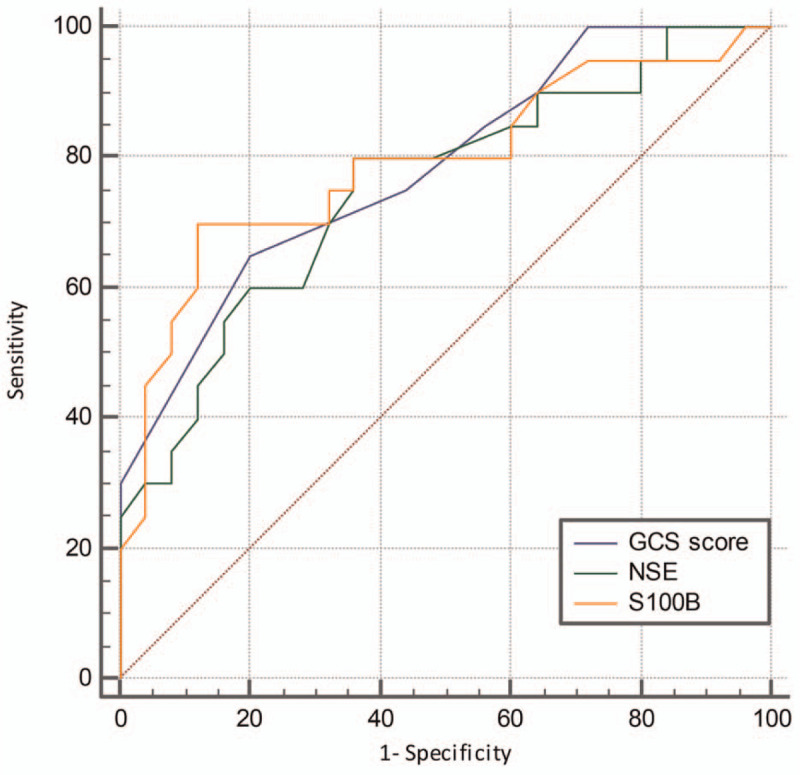
ROC curves of NSE, S100B, and GCS score to predict coma duration ≥ 72 hours.

The AUC of NSE to predict coma duration ≥ 72 hours was 0.754 [95% confidence interval (95% CI): 0.603–0.870]; the AUC of S100B to predict coma duration ≥ 72 hours was 0.791 (95% CI: 0.644–0.898), and the AUC of GCS score to predict coma duration ≥ 72 hours was 0.785 (95% CI: 0.637–0.893). Pairwise comparisons did not show significant differences among the 3 groups (all *P* > .05) (Fig. 2, Table 3).

**Table 3:** Clinical values of NSE, S100B, and GCS score in predicting coma duration ≥ 72 h.

	Cut-off value	Sensitivity	Specificity	Positive likelihood ratio	Negative likelihood ratio
NSE, μg/L	>13	80.00 (95% CI: 56.3–94.3)	64.00 (95% CI:87.7–99.6)	2.22	0.31
S100B, μg/L	>0.43	70.00 (95% CI: 45.7–88.1)	88.00 (95% CI: 68.8–97.5)	5.83	0.34
GCS score	≤5	65.00 (95% CI: 40.8–84.6)	80.00 (95% CI: 59.3–93.2)	3.25	0.44

## 4 Discussion

ACOP is commonly observed in clinic with a high occurrence rate. CO is a colorless, odorless gas, which has a high affinity to hemoglobin. The affinity of CO to hemoglobin is 240 times higher than oxygen, and its dissociation rate is only 1/3600 that of oxygen. The formation of carboxyhemoglobin reduces the blood's oxygen-carrying capacity and prevents the release of oxygen from oxygenated hemoglobin, thus resulting in serious hypoxemia. CO has a toxic effect on the tissues and cells of the whole body, particularly the cerebral cortex and brain neurons. Inflammation, demyelination of white matter, lipid peroxidation, cytotoxicity/angiogenic edema, and apoptosis are considered pathogenetic mechanisms of brain injury caused by CO poisoning.^[9]^ Patients with moderate to severe ACOP often suffer from impaired consciousness, combined with the risk for aspiration, lung infection, and pressure sores. Accurately predicting patients’ coma duration is very important for evaluating the disease conditions and preventing the occurrence of complications. Some studies have suggested that diffusion tensor imaging (DTI) can be used to detect the damage of gray matter nuclei caused by ACOP and quantify the degree of hypoxic brain damage.^[10]^ At present, there are no specific and sensitive biochemical indicators that may reflect the degree of brain injury and prognosis in patients with ACOP. This study explored the correlation and predictive value of NSE and S100B protein on coma duration in patients with ACOP.

NSE is a glycolytic enzyme that is mainly expressed in neurons and glial cells. It is also found in neuroendocrine cells, neuroendocrine tumors,^[11]^ and red blood cells (false positive due to hemolysis).^[12]^Its half-life is approximately 24 to 30 hours.^[13]^ NSE has been applied as a biomarker for the differential diagnosis of small cell lung cancer.^[14]^ It also has a certain predictive value for the prognosis of patients with severe traumatic brain injury.^[15]^ Previous animal studies have found that hemorrhagic shock or femoral fractures, rather than traumatic brain injury, can also cause an increase in NSE.^[16]^

S100B protein is an acidic calcium-binding protein composed of α and β subunits that have an essential role in maintaining calcium homeostasis.^[17]^ S100B is mainly expressed in astrocytes, mature oligodendrocytes, and renal epithelial cells. Healthy people have very low baseline serum S100B levels (approx. 0.05 μg/L). Because astrocytes are as sensitive to hypoxia as neurons, the increase of S100B protein can indirectly reflect nerve function damage. The half-life of S100B is relatively short (about 30 minutes),^[18]^ so the continuous rising of S100B may be a sign of persistent brain injury. As it does not pass through an intact blood--brain barrier (BBB), S100B has been applied as a marker of enhanced BBB permeability.^[19]^ However, S100B may also be found outside nerve tissues, such as muscle, chondrocytes,^[20]^ and adipocytes,^[21]^ which can confuse the results in some patients, such as those with cardiac arrest receiving extrathoracic compressions.

Kim et al^[22]^ found that CO exposure duration > 5 hours [AOR (adjusted odds ratio) 7.082; 95% CI: 3.463–15.014; *P* < .001], abnormal white blood cell counts (AOR 2.568; 95% CI: 1.188–5.700; *P* = .02), and abnormal creatinine concentration (AOR 2.667; 95% CI: 1.110–6.605; *P* = .03) were predictive factors of brain injury with impaired consciousness caused by ACOP. The predictive value of CO exposure duration was the highest (AUC = 0.815), and the optimal cut-off value was 5 hours. This study found a correlation between CO exposure duration and impaired consciousness in patients with ACOP. The CO exposure duration in Coma < 72 h group and Coma ≥ 72 h group were longer than that in the Awake group. In addition, there was no significant difference between the 2 Coma groups. However, the value of biomarkers, such as NSE and S100B protein in patients with ACOP and impaired consciousness, was not evaluated. Another study showed that serum lactic acid levels, serum anion gap, and serum S100B protein levels could be used as predictors of delayed neuropsychologic sequelae (DNS) in ACOP patients and can help to identify DNS in ACOP patients at an early stage.^[23]^

This study revealed significant differences in GCS score, NSE, and S100B protein levels between the Coma < 72 h group and the Awake group, and between Coma ≥ 72 h group and the Awake group. Meanwhile, the GCS score was lower, and NSE and S100B protein levels were higher in Coma ≥ 72 h group than those in Coma < 72 h group, and the differences were statistically significant. These data indicated that GCS score, NSE, and S100B protein levels were correlated with the duration of consciousness impairment. Meanwhile, we also found a negative correlation between S100B and GCS score (*r*_s_ = -0.590, *P* < .01), which was consistent with the results of Cakir et al.^[24]^ Yet, the negative correlation between NSE and GCS score (*r*_s_ = -0.583, *P* < .01) was different, which was related to the sample size and the time of sample collection. The levels of NSE and S100B protein were also affected by a variety of intracranial and extracranial factors and detection methods.^[25]^Although the levels of NSE and S100B were correlated with the degree and duration of consciousness impairment in patients with ACOP, they were not significantly different from the GCS score in predicting coma duration ≥ 72 hours in the ROC curve analysis (AUC of the 3 were 0.754, 0.791, and 0.785, respectively, *P* > .05). However, considering the limitations of GCS score in clinical application, such as drinking history, seizures, use of sedatives, electrolyte abnormalities, blood glucose abnormalities, past nervous system diseases, trauma history, and doctors’ subjective judgment, the results of NSE and S100B levels may be more objective and more reliable. Compared with NSE, S100B protein seemed to be more useful in predicting the prognosis of neurological function after cardiopulmonary resuscitation due to its higher specificity.^[26]^ The NSE gradually reached the peak at 72 hours after cardiopulmonary resuscitation^[27]^, which obviously lagged behind the S100B protein, thus suggesting that NSE fell behind S100B protein in the early evaluation of neurological function. However, in this study, we did not detect a statistical difference between the 2.

In conclusion, the NSE and S100B protein levels were significantly correlated with the degree of impaired consciousness in patients with ACOP, and they had the same clinical value in predicting coma duration ≥ 72 hours.

This study has a few limitations. First, the sample size was small. Therefore, the clinical value of the cut-off value needs to be verified by clinical studies with a larger sample size. Second, the NSE and S100B protein levels were measured only at a 1-time point. Further study should be conducted to investigate the changes over time and the serial cut-off values in groups according to the outcome. Third, no healthy controls were included in this study, and there was no comparison with patients with ACOP. Dynamic observation of clinical work changes may be of more clinical value than specific values, which also needs to be proven by further research.

## Acknowledgments

We would like to thank Litao Zhang, Jing Zhao, Qingqing Hao, Xin Xu, Hu Han, Jianguo Li for their assistance and valuable discussion.

## Author contributions

**Conceptualization:** Jing Zhao.

**Data curation:** Qingqing Hao.

**Methodology:** Xin Xu.

**Project administration:** Hu Han.

**Writing – original draft:** Litao Zhang.

**Writing – review & editing:** Jianguo Li.
